# Epidemiology of Brain and Other CNS Tumors

**DOI:** 10.1007/s11910-021-01152-9

**Published:** 2021-11-24

**Authors:** Quinn T. Ostrom, Stephen S. Francis, Jill S. Barnholtz-Sloan

**Affiliations:** 1 Department of Neurosurgery, Duke University School of Medicine, Durham, NC USA; 2 Department of Neurological Surgery, Division of Neuro and Molecular Epidemiology, University of California, San Francisco, CA USA; 3 Trans-Divisional Research Program, Division of Cancer Epidemiology and Genetics, and Center for Biomedical Informatics and Information Technology, National Cancer Institute, Bethesda, MD USA

**Keywords:** Brain and other CNS tumors, Epidemiology, Risk factor, Incidence, Survival

## Abstract

**Purpose of Review:**

Brain and other central nervous system (CNS) tumors, while rare, cause significant morbidity and mortality across all ages. This article summarizes the current state of the knowledge on the epidemiology of brain and other CNS tumors.

**Recent Findings:**

For childhood and adolescent brain and other CNS tumors, high birth weight, non-chromosomal structural birth defects and higher socioeconomic position were shown to be risk factors. For adults, increased leukocyte telomere length, proportion of European ancestry, higher socioeconomic position, and HLA haplotypes increase risk of malignant brain tumors, while immune factors decrease risk.

**Summary:**

Although no risk factor accounting for a large proportion of brain and other CNS tumors has been discovered, the use of high throughput “omics” approaches and improved detection/measurement of environmental exposures will help us refine our current understanding of these factors and discover novel risk factors for this disease.

## Introduction

Brain and other CNS tumors, while rare, cause significant mortality and morbidity across all ages. Despite decades of research on the etiology of brain and other CNS tumors, no risk factor accounting for a large proportion of cases has been identified. Brain and other CNS tumors are unique in that they are histologically complex, with over 100 types as listed by the World Health Organization International Classification of Diseases Oncology [1] and they display many of the well know Hallmarks of Cancer [2, 3] with dysregulated cell growth, metabolism, etc. However, with the use of novel high throughput “omics” approaches our understanding of causes and risk factors for brain and other CNS tumor continues to be refined and grow. In this review, we describe current and up to date knowledge about causes and risk factors for brain and other CNS tumors in children/adolescents and adults.

## Updates on Causes and Risk Factors for Brain and Other CNS Tumors in Children and Adolescents

**Fig. 1:**
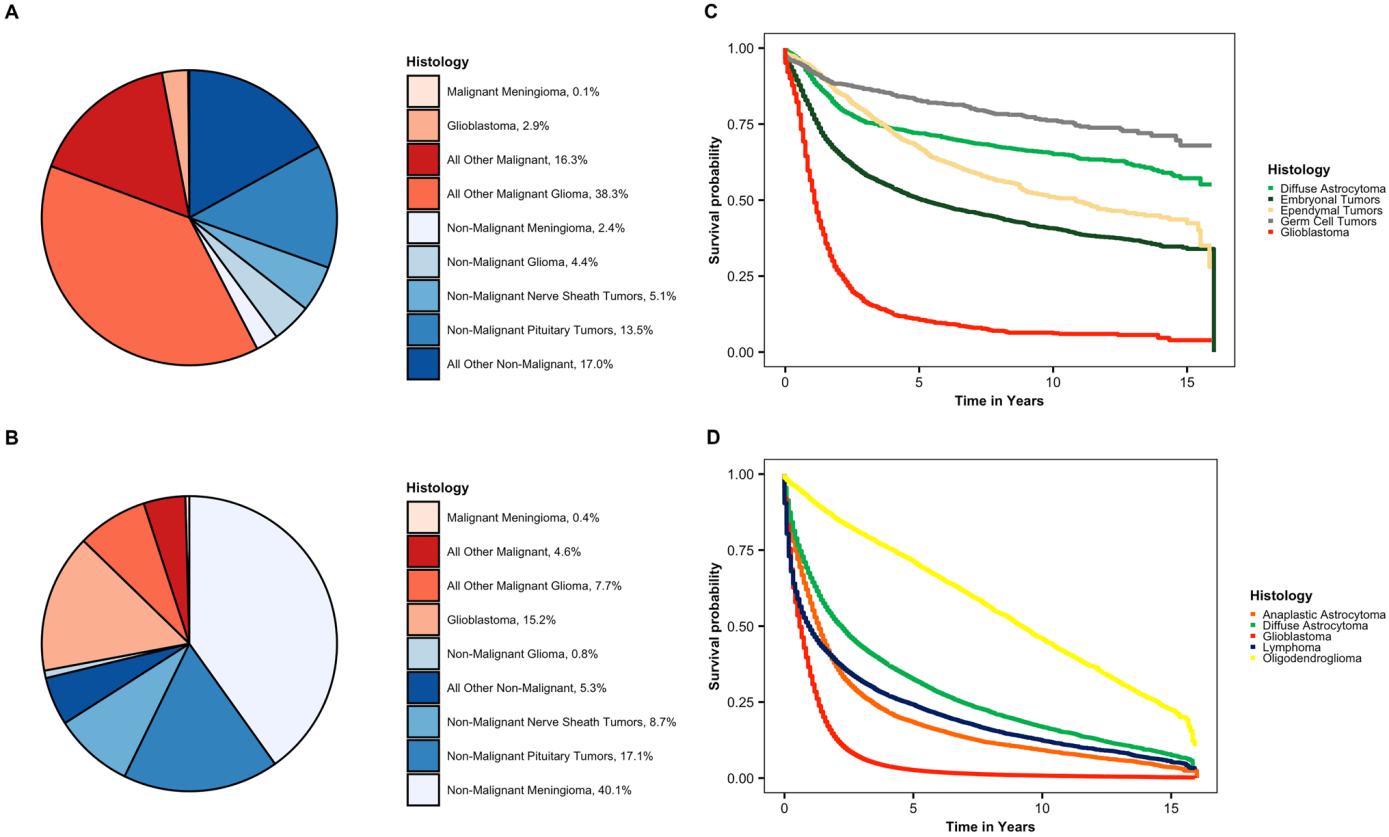
Incidence and survival for primary brain and other CNS tumors by age group, behavior and histology (CBTRUS incidence: data provided by CDC’s National Program of Cancer Registries (NPCR) and NCI’s Surveillance, Epidemiology and End Results (SEER) Program, 2013–2017; NPCR Survival Analytic file (2001–2016)), distribution of primary brain and other CNS tumors by behavior for **a** children (0–19 years), and **b** adults (20 years and older); CBTRUS: data provided by CDC’s National Program of Cancer Registries and NCI’s Surveillance, Epidemiology and End Results Program, 2013–2017; Kaplan–Meier survival curves for the five most common histologies within **c** children (0–19 years), and **d** adults (20 years and older); National Program of Cancer Registries SEER*Stat Database: NPCR Survival Analytic file (2001–2016).*Percentages may not add up to 100% due to rounding. “All Other Malignant” includes histologies with ICD − O − 3 behavior code of /3 from choroid plexus tumors, neuronal and mixed neuronal − glial tumors, tumors of the pineal region, embryonal tumors, nerve sheath tumors, mesenchymal tumors, primary melanocytic lesions, other neoplasms related to the meninges, lymphoma, other hematopoietic neoplasms, germ cell tumors, cysts and heterotopias, tumors of the pituitary, craniopharyngioma, hemangioma, neoplasm unspecified, and all other. “All Other Non-Malignant” includes histologies with ICD − O − 3 behavior code of /0 or /1 from neuronal and mixed neuronal − glial tumors, tumors of the pineal region, embryonal tumors, other tumors of cranial and spinal nerves, mesenchymal tumors, primary melanocytic lesions, other neoplasms related to the meninges, other hematopoietic neoplasms, germ cell tumors, cysts and heterotopias, craniopharyngioma, hemangioma, neoplasm unspecified, and all other

Brain and other CNS tumors the most common cancer in children diagnosed at 0–14 years old and the second most common cancer in adolescents diagnosed at 15–19 years old [4••]. In particular, the incidence of brain and other CNS tumors is highest for those 5 and younger at diagnosis. In children and adolescents, the majority of brain and other CNS tumors are malignant tumors (age-adjusted incidence of 3.55 per 100,000) while non-malignant brain and other CNS tumors are less common in this age group (age-adjusted incidence 2.60 per 100,000) [4••]. The most common malignant histologies in this age group are glioma, embryonal tumors and germ cell tumors while the most common specific non-malignant histology is tumors of the pituitary (Fig. 1a). There have been no significant changes in incidence of these tumors in this age group over the last few decades [4••, 5]. In addition, brain and other CNS tumors are the number one cause of cancer related mortality in children diagnosed at 0–14 years old and overall survival for childhood and adolescent brain and other CNS tumors varies greatly by brain and other CNS tumor histology (Fig. 1c).

Many factors, both environmental and genetic, have been studied in order to identify a factor that accounted for a large proportion of childhood and adolescent brain and other CNS tumors (as reviewed in [6•]). Unfortunately, no such factor has been identified. There are two primary risk factors for brain and other CNS tumors in children, adolescents and adults that have been well validated: single gene inherited disorders (~ 4% of childhood cases) and ionizing radiation (as reviewed in [6•, 7]). In fact, carcinogenic effects of radiation seem to be stronger in children, particularly in younger children, and show a clear dose response relationship [8, 9]. Few genetic association studies have been performed in childhood brain and other CNS tumors and therefore our knowledge about genetic risk factors for these tumors in this age group is very limited. Some candidate gene studies have been performed and provide some evidence for shared genetic risk factors for brain and other CNS tumors between age groups (as reviewed in [6•]). Some recent work in childhood ependymoma suggests that European ancestry is associated with higher risk of a childhood ependymoma [10] and that genetic risk for longer telomere length was associated with a higher risk of ependymoma in children and adolescents aged 12–19 but not for those younger than 12 years old at diagnosis [11•].

Some of the newest environmental risk factors to be studied in relation to risk for childhood and adolescent brain and other CNS tumors are birth weight and non-chromosomal structural birth defects. There is reasonably consistent evidence that higher birth weight is associated with a higher risk of childhood brain and other CNS tumors as provided by 3 large meta-analyses [12–14]. Georgakis et al. performed a systematic review and meta-analysis and showed that birth weight > 4000 g was associated with in increased risk of a childhood brain and other CNS tumor (Odds Ratio 1.14, 95% confidence interval (1.08–1.20); higher risk for astrocytoma and embryonal tumors and non-significant for ependymoma [12]. Dahlhaus et al. performed a meta-analysis and showed that high birth weight (> 4000 g) increased the risk of astrocytoma and medulloblastoma and not for ependymoma [13]. However, Bailey et al. pooled data from multiple population-based case–control studies in France and found no association between birth weight and childhood brain and other CNS tumor risk [14].

Non-chromosomal structural birth defects are a strong and consistent risk factor for childhood cancers in general [15–17]; these findings were most pronounced in young children, aged 5 years or younger with cancer [18, 19]. For brain and other CNS tumors, ~ 7% of childhood brain and other CNS tumors are attributable to these defects [15–17]. Previous studies had suggested ~ twofold increased risk of childhood brain and other CNS tumor associated with a birth defect [18–21]. However, a very recent study using records from 10 million live births showed that particularly for children with a defect of the central nervous system or other neurological anomaly they are at a higher risk of development of a brain and other CNS tumor, with hazard ratios as high as 10 [17].

## Updates on Causes and Risk Factors for Brain and Other CNS Tumors in Adults

Brain and other CNS tumors are the 8th most common cancer in adults 40 + [4••]. The majority of brain and other CNS tumors diagnosed in adults 20 + years old are non-malignant tumors (age-adjusted incidence of 22.38 per 100,000) while malignant brain and other CNS tumors are less common in this age group (age-adjusted incidence 8.5 per 100,000) [4••]. The most common malignant histology in adult is glioma, while the most common specific non-malignant histologies are meningioma and tumors of the pituitary (Fig. 1b). There have been no significant changes in incidence of glioma in this age group over the last few decades [4••, 5]. Malignant brain and other CNS tumors are the 6th most common cause of cancer death in adults 40 + years old in the USA [4••]. Overall, survival for adult brain and other CNS tumors varies greatly by brain and other CNS tumor histology (Fig. 1d).

Numerous environmental exposures have been evaluated as potential risk factors for brain and other CNS tumors in adults, but the only consistent risk factor that has been identified is exposure to high-dose ionizing radiation [22]. For meningioma, the excess relative risk (ERR) associated with one Gy of exposure to ionizing radiation was 4.63, while the ERR associated with glioma was 1.98. History of respiratory allergies has been consistently associated with decreased risk of glioma [23]. Due to the rarity of this level of radiation exposure, this does not account for the vast majority of brain tumor incidence.

Many environmental risk factors are still under investigation, though these have mixed or null results of association with brain and other CNS tumors. One of the most thoroughly investigated is cellular phones due to their frequent use globally. Cellular phones emit radiofrequency fields (RF), which were classified as a possible carcinogen by the International Agency for Research on Cancer (IARC) in 2011 [24]. The majority of epidemiological studies since the publication of the IARC report have found no significant associations between cellular phone use and risk of any type of brain and other CNS tumor. Extremely low frequency magnetic fields (ELFs) have also been studies extensively in relation to brain and other CNS tumor risk. The INTEROCC consortium was formed to evaluate the association between ELF and brain and other CNS tumors, and did not find an association with lifetime cumulative occupational exposure to ELF [25]. Power lines are another source of EMF exposure that have been investigated in relation to brain and other CNS tumor risk. A recent case–control study found a significant association between the highest level of estimated ELF from power lines and increased risk of brain and other CNS tumors, and glioma in particular [26]. More investigation is necessary to confirm this association. Other non-radiation occupational exposures have also been studied extensively in relation to risk for brain and other CNS tumors, and to date none have been consistently associated with risk of brain and other CNS tumors [6•].

While the vast majority of brain and other CNS tumors occur in individuals without a known cancer syndrome, ~ 5–10% have a family history of brain and CNS tumor [27]. There are numerous mendelian cancer syndromes that affect risk of brain and other CNS tumors, including neurofibromatosis types I and II, tuberous sclerosis, and Li Fraumeni syndrome (as reviewed [6•]; Table 1). Due to the lack of known environmental risk factors, investigations into common inherited genetic polymorphisms have been conducted to identify genetic risk factors in individuals with no family history. The majority of these studies have focused on glioma, which is responsible for the vast majority of deaths due to malignant brain and other CNS tumors. In total, these have identified 25 single nucleotide polymorphisms (SNPs) associated with risk for glioma. The risk conferred by these variants is histology specific. There are 11 risk SNPs for glioblastoma and 19 risk SNPs for non-glioblastoma, where 5 SNPs are shared between these two broad glioma types [28••] (Table 1). The function of many gliomas associated SNPs are currently unknown, though some are part of known oncogenic pathways. The most common pathway identified as conferring risk in glioma are those associated with telomere maintenance, including risk variants near *TERT* and *RTEL1*. Many of these SNPs have further molecular subtype associations ([29•]; Table 1). Several candidate SNP studies have been conducted in East Asian populations, which have found novel association loci for glioma as well as validated those discovered in European-ancestry populations, including loci in *TERC*, *TERT*, *EGFR*, and *PHLDB1* [30, 31] (Table 1). The only GWAS of glioma in an East Asian population confirmed associations near *TERT*, *PHLDB1* and *RTEL1*, and identified two new variants [32•] (Table 1).

**Table 1:** Genes implicated in inherited and sporadic brain tumor risk by chromosomal position (as reviewed in in [6•])

Chromosomal location	Gene	Associated tumor type	Mendelian associations disorder/syndrome (OMIM ID)	Single SNP associations from genome-wide association studies
2p16.3	*MSH6*	Medulloblastoma, glioma, glioblastoma,	Lynch syndrome (120435), Biallelic mismatch repair deficiency, constitutional MMR *deficiency* Mismatch repair deficiency syndrome (276300)	*None*
2p21-p16.3	*MSH2*	Medulloblastoma, glioma, glioblastoma,	Lynch syndrome (120435), Biallelic mismatch repair deficiency, constitutional MMR *deficiency* Mismatch repair deficiency syndrome (276300)	*None*
2q33.3	*C2orf80*	Lower grade glioma	*None*	rs7572263
2q33.3	*IDH1*	Glioma	Ollier disease	*None*
3p14.1	*LRIG1*	Lower grade glioma	*None*	rs11706832
3p21.1	*BAP1*	Meningioma	BAP1 tumor predisposition syndrome (614327)	*None*
3p22.2	*MLH1*	Medulloblastoma, glioma, glioblastoma,	Turcot’s syndrome type 1 Lynch syndrome (120435), Biallelic mismatch repair deficiency, constitutional MMR deficiency Mismatch repair deficiency syndrome (276300)	*None*
3p25	*VHL*	Hemangioblastoma	Von Hippel-Lindau syndrome (193300)	*None*
3q26.2	*TERC*	All glioma	*None*	rs1920116
5p13.3	*DROSHA*	Pineoblastoma, pituitary blastoma	*DICER1* syndrome	*None*
5p15.33	*TERT*	All glioma	*None*	rs10069690
		Astrocytoma	*None*	rs2853676
5q21	*APC*	Medulloblastoma, glioma	Familial adenomatous polyposis (FAP, 175100), Turcot’s syndrome type 2	*None*
7p11.2	*EGFR*	All glioma	*None*	rs2252586
		Glioblastoma	*None*	rs11979158; rs730437; rs1468727
7p22.1	*PMS2*	Medulloblastoma, glioma, glioblastoma,	Turcot’s syndrome type 1 Lynch syndrome (120435), Biallelic mismatch repair deficiency, constitutional MMR deficiency Mismatch repair deficiency syndrome (276300)	*None*
8p12	*RECQL2*	Meningioma	Werner syndrome (277700)	*None*
8q24.21	*CCDC26*	Lower grade glioma, in particular IDH-mutant tumors	*None*	rs55705857
9p21.3	*CDKN2A*	Glioma	Melanoma-neural system tumor syndrome (155755)	*None*
	*CDKN2B-AS1*	Lower grade glioma, in particular WHO grade II-IV astrocytic tumors	*None*	rs4977756
9q22.3	*PTCH1*	Medulloblastoma, meningioma	Gorlin’s syndrome (nevoid basal cell carcinoma)	*None*
9q34.14	*TSC1*	Giant cell astrocytoma	Tuberous sclerosis (TSC) (191100, 613254)	*None*
10p12.31	*MIR4675*, *NEBL*	Pituitary adenoma	*None*	rs2359536
	*MLLT10*	Meningioma	*None*	rs11012732
10q21.1	*PCDH15*	Pituitary adenoma	*None*	rs10763170
10q23.31	*PTEN*	Cerebellar gangliocytoma, meningioma	Cowden syndrome 1 (158350)	*None*
10q24.32	*SUFU*	Meningioma	Familial meningiomatoses (607174)	*None*
10q24.33	*OBFC1*	Lower grade glioma	*None*	rs11598018
10q25.2	*VTI1A*	Lower grade glioma	*None*	rs11599775
11p15.5	*RIC8A*	Meningioma	*None*	rs2686876
11q13.1	*MEN1*	Pituitary prolactinoma, meningioma	Multiple endocrine neoplasia, type 1 (131100)	*None*
11q13.2	*AIP*	Pituitary adenomas	Pituitary adenoma predisposition (102200)	*None*
11q14.1	Intergenic	Glioblastoma	*None*	rs11233250
11q21	*MAML2*	Lower grade glioma	*None*	rs7107785
11q22.3	*ATM*	Astrocytoma and medulloblastoma	Ataxia-telangiectasia (208900)	*None*
11q23.2	*PHLDB1*	All glioma	*None*	rs648044; rs17748; rs2236661; rs494560
		All glioma	*None*	rs494560
		Lower grade glioma, in particular IDH-mutant gliomas	*None*	rs498872
12p11.23	*STK38L*	All glioma	*None*	rs10842893
12q21.2	Intergenic	Lower grade glioma	*None*	rs1275600
13q12.13	*CDK8*	Pituitary adenoma	*None*	rs17083838
13q14	*RB1*	Retinoblastoma, pineoblastoma, Malignant glioma	Retinoblastoma	*None*
14q12	*AKAP6*	Lower grade glioma	*None*	rs10131032
14q32.13	*DICER1*	Pineoblastoma, pituitary blastoma	*DICER1* syndrome	*None*
15q21.3	*RAB27A*	All glioma	*None*	rs4774756
15q24.2	*ETFA*	Lower grade glioma	*None*	rs1801591
15q26.1	*IDH2*	Glioma	Ollier disease	*None*
16p13.3	*CREBBP*	Medulloblastoma, oligodendroglioma, and meningioma	Rubinstein-Taybi syndrome (180849)	*None*
16p13.3	*RHBDF1*	Glioblastoma	*None*	rs2562152
		Lower grade glioma	*None*	rs3751667
	*TSC2*	Giant cell astrocytoma	Tuberous sclerosis (TSC) (191100, 613254)	*None*
16q12.1	*HEATR3*	Glioblastoma	*None*	rs10852606
16q24.3	*FANCA*	Medulloblastoma	Fanconi anemia (227650)	*None*
17p13.1	*TP53*	All glioma	Li-Fraumeni syndrome (151623)	rs78378222
17q11.2	*NF1*	Astrocytoma, schwannomas, optic nerve glioma	Neurofibromatosis 1 (NF1) (162200)	*None*
17q21.2	*SMARCE1*	Meningioma	Familial meningiomatoses (607174)	*None*
17q24.2	*PRKAR1A*	Pituitary adenomas	Carney complex (160980)	*None*
1p31.3	*RAVER2*	Glioblastoma	*None*	rs12752552
1q32.1	*MDM4*	Lower grade glioma	*None*	rs4252707
1q44	*AKT3*	Lower grade glioma	*None*	rs12076373
20q13.33	*RTEL1*	All glioma	*None*	rs6010620
22q11.23	*SMARCB1*	Meningioma	Familial meningiomatoses (607174)	*None*
22q12.1	*MN1*	Meningioma	Familial meningiomatoses (607174)	*None*
22q12.2	*NF2*	Acoustic neuromas, meningiomas, Ependymoma	Neurofibromatosis 2 (NF2) (101000)	*None*
22q13.1	*PDGFB*	Meningioma	Familial meningiomatoses (607174)	*None*
	*SLC16A8*	Glioblastoma	*None*	rs2235573
22q13.2	*EP300*	Medulloblastoma, oligodendroglioma, and meningioma	Rubinstein-Taybi syndrome (180849)	*None*

### Ancestry and Brain Tumor Risk

Genetic studies have also been conducted in other brain and other CNS tumor types. In European ancestry populations, two SNPs have been identified as affecting risk for meningioma [33•] (Table 1), while two SNPs have been identified for primary CNS lymphomas [34•] (Table 1). In individuals of East Asian ancestry, three SNPs have been identified as increasing risk in pituitary adenoma [35]. Genetic factors other than specific SNPs have also been associated with risk of developing a brain tumor. Increased leukocyte telomere length (LTL) has been associated with increased risk of both glioma and meningioma [36, 37]. In addition to individual level variation in LTL, analysis of glioma samples has demonstrated that these tumors have significantly longer telomere length as compared to other cancers [38]. Malignant brain tumor incidence is highest in countries with primarily European-ancestry populations (such as Europe, the USA and Canada), and in white non-Hispanics in the USA [6•, 39]. Similar to associations identified with pediatric tumors, increased overall European-ancestry has been detected in African American and Hispanic glioma cases as compared to controls [40•].

### Immune Related Factors: Viruses, Allergy, and HLA

Several infections have been epidemiologically evaluated in glioma. Members of the polyomavirus family including BK, JC, and SV40 have been inconsistently associated with glioma risk [41, 42]. Members of the family *herpesviridae* have been evaluated in multiple studies with inconsistent results. The herpesvirus’s Epstein-Barr virus, herpes-simplex 1/2, has been extensively evaluated in human cancers; yet, the evidence in central nervous system tumors is contradictory [43, 44]. Cytomegalovirus (CMV) was associated with glioma where serologic investigations into risk/survival and the presence of CMV within tumors have again provided inconsistent evidence of a causal link between CMV and glioma development [45–48]. However, recently two anti-CMV therapeutics have provided evidence of increased patient survival after receiving valganciclovir or a pp65 based treatment [49, 50]. Those observations and mechanistic studies have bolstered a theory of CMV as an ‘oncomodulator’ in glioma, where CMV may not necessarily be involved in the initiation of glioma but may play a role in tumor growth and immune evasion [51•]. The most recently associated infection with glioma risk is not a virus but a protozoan, *toxoplasma gondii* (*T. gondii*). In a relatively small study of serum samples from two separate cohorts antibodies to *T. gondii* were significantly associated (OR: 2.70; 95% CI: 0.96–7.62; OR: 1.32, 95% CI: 0.85–2.07) with glioma risk before diagnosis, eliminating reverse causation biasing the association [52]. Further serologic studies examining *T. gondii* are needed.

The only consistently associated infection tied to glioma risk is the herpesvirus varicella zoster virus (VZV), the nearly ubiquitous virus that causes chickenpox and shingles [53]. Serologic studies of VZV antigens have also shown a similar reduction in glioma risk [54, 55]. In a large international meta-analysis of self-report VZV infection reported from 8704 cases included in the Glioma International Case Control Study, infection with VZV conferred a 20% reduced risk of glioma [56]. Although the mechanism remains a mystery, it has been hypothesized that interactions between the VZV and host immune response may be mediating glioma development. Parallel to the inverse association with VZV is the observation that allergic and ectopic conditions reduce glioma risk [23]. Allergies and other atopic conditions have consistently been shown to reduce risk of brain tumors, particularly glioma (as reviewed in [6•]).

Two large international meta-analyses have also concluded that allergy and ectopic conditions reduce the risk of glioma ~ 20% [23, 57]. Measurements of serum IgE in glioma cases and controls have mirrored the questionnaire based studies showing that increased serum IgE is associated with reduced glioma risk [58, 59]. To further investigate the underlying genetic architecture of allergy and its relation to glioma risk Mendelian randomization studies have been utilized to assess the genetic basis for this association [60–62]. The results from these studies have been suggestive showing small effects of reduced risk when comparing genetically programmed allergy/atopy with glioma risk, but not conclusive and may be due to the difficulty of constructing a genetic instrument for allergy and ectopic conditions.

Studies have demonstrated a significant heritable component (32–48%) of antibody responses to many viruses and have identified multiple host genetic loci relating to immune response for a variety of viruses [63]. The hereditable component for allergic response is estimated at ~ 65% and genetic loci relating to T-cell and signal transduction [64–66]. Genetic studies of both allergy and response to infections have highlighted the human leucocyte antigen (HLA) as a powerful genetic regulator. Specific HLA alleles have been associated with glioma, though the complexity of the HLA complicates studies based on SNP array data. One of the earliest studies to investigate this was the UCSF Adult Glioma Study, with risk-increasing effects observed for B*13 and B*07 ~ C*07 haplotype, and protective effects for C*01 allele [67]. In this same study, two class I HLA alleles, A*32 and B*55, were associated with longer survival in GBM AGS patients. A*32 was also inversely associated with GBM risk in a separate population [68]. The largest recent study of using SNPs1856 glioma cases and 4955 controls, observed a 50% greater risk of glioma in heterozygous compared to homozygous carriers of the DRB1*15:01 ~ DQA1*01:02 ~ DQB1*06:02 haplotype (p < 0.002), with significant non-additive/epistatic effects [69]. Intriguingly, this haplotype is associated with susceptibility to multiple autoimmune conditions, and antibody response to EBV and VZV antigens [70, 71], and a new analysis suggested that history of auto-immune disease may also decrease risk of developing a glioma [72•]. Recent analyses of expression of immune cell populations using LD score regression showed that the genomic architecture of T cells, NK cells, and myeloid cells is inversely correlated with glioma and may be mediating glioma predisposition [72•]. New approaches to categorizing immune cells in tumors include traditional immunohistochemistry-based approaches [73] and novel methylation based analyses to de-convolute cell types [74•]; both of these approaches seek to stratify tumor types based on tumor infiltrating immune cells. Recent studies show that methylation derived neutrophile to lymphocyte ratios less than 4.0 were associated with significantly decrease survival times (HR 2.02, 95% CI, 1.11–3.69) [75]. Further research examining the interaction between genetic loci, blood cell proportions and their relationship to allergy/infections are required to understand the complex involvement to glioma risk.

### Socioeconomic Position

Mounting evidence from diverse studies suggests that higher socioeconomic position (SEP) is associated with an increased risk of adult CNS tumors when compared to individuals with a lower SEP [76–79, 80••]. An analysis of SEER data showed a significant relationship between the first quartile versus the second third, and fourth quartiles of county level income revealing a 10%, 11%, and 14% higher risk of glioma respectively [77]. A recent analysis of SEER data showed that the increased risk associated with higher SEP is primarily in non-Hispanic whites [80••]. Additionally, two recent registry-based studies of childhood CNS malignancies suggest that this relationship appears to not only exist in adult CNS tumors but also in childhood CNS tumors, where studies in both California and Denmark show similar effects in various measures of SEP [81•, 82•]. Possible explanations include a diagnostic bias where tumors in patients with lower SEP may go unreported; yet, the accuracy of surveillance and the magnitude of the effect suggest that this bias alone does not alone account for the association. Another explanation is an unidentified risk factor that is associated with higher SEP, possibly related to the ‘hygiene hypothesis’ [83] where immune exposures relating to allergy and infection maybe altered according to SEP.

## Conclusions

Although no risk factor accounting for a large proportion of brain and other CNS tumors has been discovered, there are multiple directions that can be taken to add to our understanding of risk for brain and other CNS tumors. Specifically, the use of high throughput “omics” approaches, improved detection/measurement of environmental exposures, expansion to more diverse populations, synergy between germline and somatic variants, and incorporation of all types of clinical data to comprehensively study this disease (such as imaging). These novel directions will help us refine our current understanding of these factors and discover novel risk factors for this disease.
